# Development and Validation of a Computed Tomography–Based Radiomics Signature to Predict Response to Neoadjuvant Chemotherapy for Locally Advanced Gastric Cancer

**DOI:** 10.1001/jamanetworkopen.2021.21143

**Published:** 2021-08-19

**Authors:** Wei Wang, Ying Peng, Xingyu Feng, Yan Zhao, Sharvesh Raj Seeruttun, Jun Zhang, Zixuan Cheng, Yong Li, Zaiyi Liu, Zhiwei Zhou

**Affiliations:** 1 Department of Gastric Surgery, Sun Yat-sen University Cancer Center, Guangzhou, People’s Republic of China; 2 State Key Laboratory of Oncology in South China, Collaborative Innovation Center for Cancer Medicine, Guangzhou, People’s Republic of China; 3 Department of General Surgery, Guangdong Provincial People’s Hospital, Guangdong Academy of Medical Sciences, School of Medicine, South China University of Technology, Guangzhou, People’s Republic of China; 4 Department of Stomach Surgery, Cancer Hospital of China Medical University (Liaoning Cancer Hospital & Institute), Shenyang, People’s Republic of China; 5 Department of Radiology, Guangdong Provincial People’s Hospital, Guangdong Academy of Medical Sciences, School of Medicine, South China University of Technology, Guangzhou, People’s Republic of China

## Abstract

**Importance:**

Neoadjuvant therapies have been shown to decrease tumor burden, increase resection rate, and improve the outcomes among patients with locally advanced gastric cancer (GC). However, not all patients are equally responsive; therefore, differentiating potential respondents from nonrespondents is clinically important.

**Objective:**

To use pretreatment computed tomography (CT)–pixelated feature-difference extraction techniques to identify diagnostically relevant features that could predict patients’ response to neoadjuvant chemotherapy at diagnosis.

**Design, Setting, and Participants:**

This multicenter cohort study included patients with locally advanced GC who were treated from January 2010 to July 2017 at 2 hospitals in southern China (training cohort) and 1 hospital in northern China (external validation cohort). Their clinicopathological data, pretreatment CT images, and pathological reports were retrieved and analyzed. Data analysis was conducted from December 2017 to May 2021.

**Exposures:**

All patients underwent 2 to 4 cycles of fluorouracil in combination with a platinum-based neoadjuvant chemotherapy regimen. All gastrectomies were performed according to the Japanese Classification of Gastric Carcinoma (14th edition) guidelines.

**Main Outcomes and Measures:**

Reliability of clinicopathological and radiomics-based features were assessed with area under receiver operating characteristic curve (AUC) and Mann-Whitney *U* test.

**Results:**

A total of 323 patients (242 [74.9%] men; median [range] age, 58 [24-82] years) were included in the study, with 250 patients (77.4%) in the training cohort and 73 (22.6%) in the validation cohort. The baseline pretreatment characteristics of the training and validation cohorts were well-balanced. The number of respondents in the training and validation cohort was 122 (48.8%) and 40 (54.8%), respectively, and the number of nonrespondents was 128 (51.2%) and 33 (45.2%), respectively. No clinicopathological variables were significantly associated with treatment response. Using radiomics, 20 low-intercorrelated features from a total of 7477 features were used to construct a radiomics signature that demonstrated significant association with treatment response. Good discrimination performance of the radiomics signature for predicting treatment response in the training (AUC, 0.736; 95% CI, 0.675-0.798) and external validation (AUC, 0.679; 95% CI, 0.554-0.803) cohorts was observed. Decision curve analysis confirmed the clinical utility of the radiomics signature.

**Conclusions and Relevance:**

In this study, the proposed radiomics signature showed potential as a clinical aid for predicting the response of patients with locally advanced GC before treatment, thereby allowing timely planning for effective treatments for potential nonrespondents.

## Key Points

**Question:**

Can computed tomography–based radiomics be used to predict patients’ response to neoadjuvant chemotherapy at diagnosis?

**Findings:**

In this cohort study of 323 patients with locally advanced gastric cancer, no pretreatment clinicopathological characteristics were associated with response or nonresponse to treatment. However, 20 radiomic features with low intercorrelation were significantly associated with treatment response and were used to create a radiomics signature with promising clinical reliability for identifying potential responders to neoadjuvant chemotherapy.

**Meaning:**

Given that no patients respond equally to neoadjuvant chemotherapy, the radiomics signature proposed in this study could guide clinicians in selecting appropriate patients for neoadjuvant chemotherapy.

## Teaser

This cohort study uses pretreatment computed tomography (CT)–pixelated feature-difference extraction techniques to identify diagnostically relevant features that could predict patients’ response to neoadjuvant chemotherapy for locally advanced gastric cancer.

## Introduction

Gastric cancer (GC) is a highly heterogeneous disease associated with a high mortality rate.^1^ Although multimodal treatments, such as gastrectomies followed by adjuvant chemotherapy (the standard of care especially in Asian countries),^2^ have improved patients’ survival outcomes,^3^ benefits for locally advanced cases have been modest. The addition of neoadjuvant therapies to standard treatment has led to an increase in pathological complete remission (pCR) and pathological partial remission (pPR) rates of the tumor, an increase in resection rates, and improvements in survival outcomes.^4,5,6,7^ Thus, neoadjuvant therapy is being increasingly used outside clinical trial settings.

However, not all patients are equally responsive to neoadjuvant therapies. Differentiating between potential responders and nonresponders is critical for timely treatments. Also, considering that pCR and pPR are confirmed through invasive histopathological interventions and mostly after surgery, creating an alternative, noninvasive method remains a major challenge. Conventional and multicombinative radiographic approaches have been explored, but their accuracies have been limited.^8,9^ On the other hand, molecular biomarkers, including serologic and radiographic markers, have shown promising results, but none have been prospectively validated for routine clinical use.^10,11^

Radiomics has shown promise for extracting noninvasive radiographic virtual biopsy biomarkers. Landmark discoveries have been observed in nasopharyngeal,^12^ lung,^13^ breast,^14^ and urinary bladder^15^ cancers, demonstrating improved predictive values of multiparametric imaging features over conventional imaging metrics. We previously developed and validated a radiomics-based nomogram to predict preoperative lymph node metastasis in patients with colorectal cancer using radiomics-derived signatures.^16^ Liu et al^17^ developed and validated an individualized radiomics model to noninvasively predict pCR in patients with advanced colorectal cancer who received neoadjuvant treatment and were able to identify patients who could avoid surgery after neoadjuvant therapy. Regarding GC, research on radiomics for predicting pretreatment responses to neoadjuvant therapy via multicenter studies with robust validation is yet to be reported. Here, using a large cohort of patients with locally advanced GC from 3 high-volume institutions, a radiomics signature was developed and externally validated for the individualized prediction of pretreatment response to neoadjuvant chemotherapy to differentiate potential respondents from nonrespondents.

## Methods

### Data Retrieval

**Figure 1.**
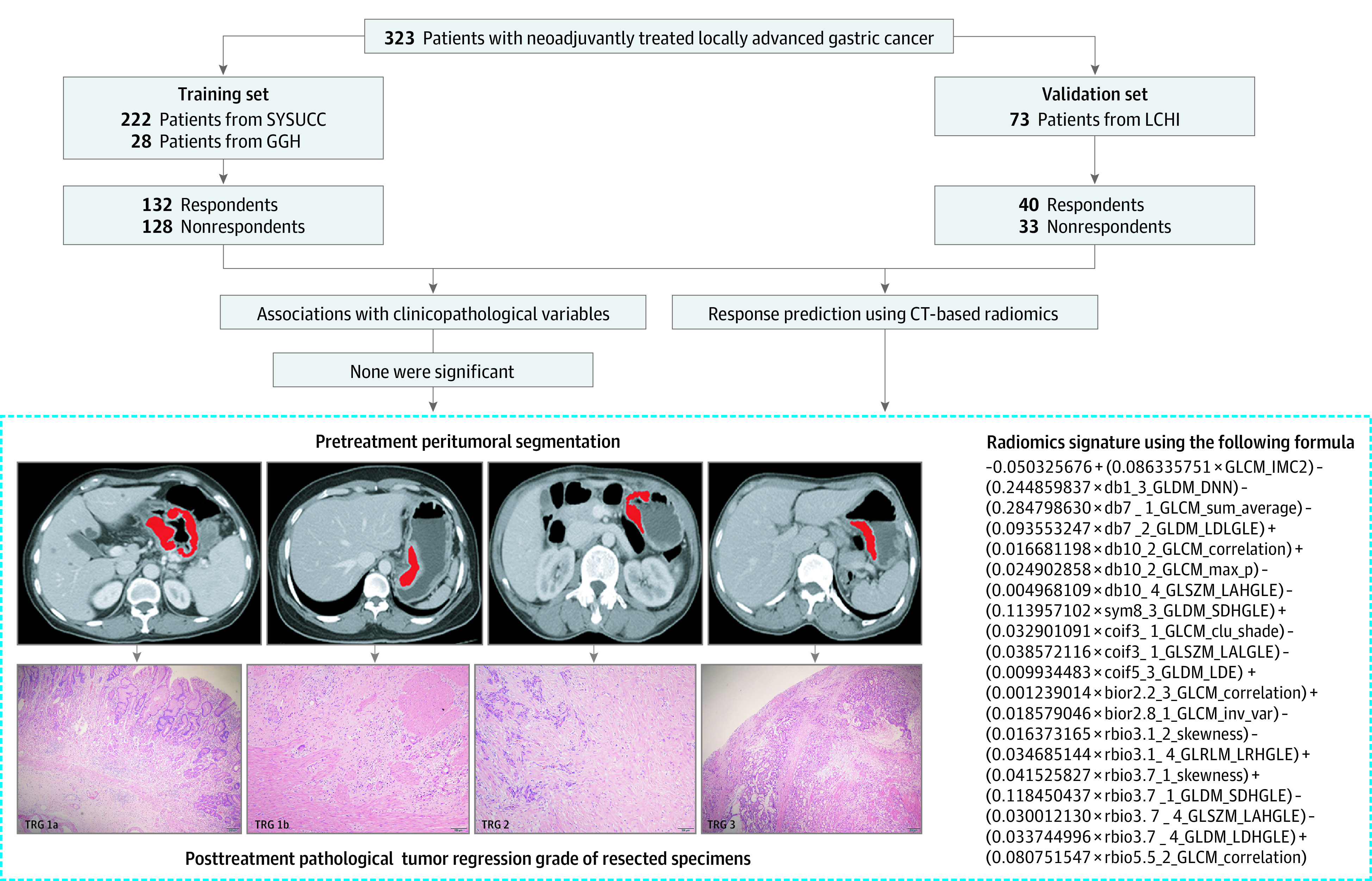
Flowchart Illustrating the Study Recruitment, Patients Categorization, and Steps for Developing the Radiomics Signature for Pretreatment Response Prediction Clu_shade indicates cluster shade; GGH indicates Guangdong General Hospital; GLCM, gray level co-occurrence matrix; IMC, informational measure of correlation; GLDM, gray level dependence matrix; DNN, dependence nonuniformity normalized; GLRLM, gray level run length matrix; GLSZM, gray level size zone matrix; LAHGLE, large area high gray level emphasis; LCHI, Liaoning Cancer Hospital and Institute; LALGLE, large area low gray level emphasis; LDE, large dependence emphasis; LDHGLE, large dependence high gray level emphasis; LDLGLE, large dependence low gray level emphasis; LRHGLE, long run high gray level emphasis; SDHGLE, small dependence high gray level emphasis; and SYSUCC, Sun Yat-sen University Cancer Center.

The demographic data, clinical characteristics, enhanced pretreatment CT images, pathological findings of biopsies before treatment and gross specimen after gastrectomy, and operative records of patients with locally advanced GC who were treated with neoadjuvant chemotherapy from January 2010 to July 2017 at 3 high volume centers were retrospectively retrieved. All patients underwent CT examination within 1 week before initiation of neoadjuvant chemotherapy. The main inclusion criteria were (1) biopsy-proven gastric adenocarcinoma; (2) nonmetastatic locally advanced GC determined by pretreatment CT examination or laparoscopic laparotomy; and (3) gastrectomy procedure after completion of neoadjuvant chemotherapy, after which tumor response was confirmed by postoperative pathologic examination. Main exclusion criteria were (1) presence of other gastric malignant neoplasms; (2) inoperable or nonresected cases (ie, exploratory laparoscopy only without gastrectomy; gastrojejunal anastomosis without tumor resection); (3) other types of neoadjuvant therapies; and (4) insufficient CT imaging quality to perform measurements (eg, due to motion artifacts). All gastrectomies were performed according to the Japanese Classification of Gastric Carcinoma (14th edition) guidelines. The 8th edition of the American Joint Committee on Cancer (AJCC) tumor-node-metastasis (TNM) classification was used for tumor staging.^18^ Patients from the Sun Yat-sen University Cancer Center (SYSUCC; Guangzhou, China) and Guangdong General Hospital (GGH; Guangzhou, China) were grouped as the training cohort, and those from Liaoning Cancer Hospital and Institute (LCHI; Liaoning, China) were grouped as the external validation cohort. The detailed enrollment process is illustrated in Figure 1.

The study protocol adhered to the regulations of the Declaration of Helsinki^19^ and was also approved by the ethics committee at each institution. All patients provided written informed consent at the time of diagnosis to use their data for scientific purposes. This study followed the Strengthening the Reporting of Observational Studies in Epidemiology (STROBE) reporting guideline for cohort studies.

### Neoadjuvant Chemotherapy Treatment and Response Assessment

The prescribed neoadjuvant chemotherapy regimen was unified as fluorouracil in combination with platinum-based chemotherapy for 2 to 4 cycles. Because the data were retrospectively retrieved, the dose prescribed to each patient depended on the treating oncologists but still adhered to the standard National Comprehensive Cancer Network guidelines for GC clinical practice at the time of treatment.

After neoadjuvant chemotherapy, all patients underwent gastrectomy. The surgically resected specimens were histopathologically assessed by expert pathologists with more than 10 years of experience in gastrointestinal cancer assessment and were blinded to the study data. The tumor regression grade (TRG) proposed by Becker et al^20^ was adopted to evaluate neoadjuvant treatment response. Briefly, tumor samples without residual tumor were defined as complete tumor regression (TRG 1a). When more than 50% of residual tumors were identified, the sample was classified as minimal or no tumor regression (TRG 3). The presence of less than 10% of residual tumor cells per tumor bed was classified as sub-total regression (TRG 1b) and 10% to 50% of residual tumor cells per tumor bed was defined as partial regression (TRG 2). Furthermore, patients with TRG 1a and 1b were considered respondents to neoadjuvant chemotherapy, while those with TRG 2 and 3 were classified as nonrespondents.

### Radiomics Feature Extraction and Reproducibility Assessment

The CT features extraction process is described in the eMethods in the Supplement. The region of interest (ROI) was manually delineated using portal venous phase CT images. The radiomics features extracted included (1) first-order statistics, (2) shape-based features, (3) texture features, and (4) wavelet features.

Three experienced radiologists were randomly assigned 30 CT images (10 patients’ images for each reader with 8, 10, and 13 years of clinical experience in abdominal cancer CT interpretation) for ROI-based feature extraction to assess intra-observer and interobserver reproducibility. Intraclass and interclass correlation coefficients (ICC) were used to assess the stability, reproducibility, and robustness of the extracted features. Features with an ICC of greater than 0.75 were considered to have agreeable reproducibility and were chosen for further analysis.

### Radiomics Feature Selection and Radiomics Signature Building

For constructing the radiomics signature, similar features with high correlations were rejected using the Spearman correlation coefficients. Feature pairs with Spearman correlation coefficients of greater than 0.9 were considered highly correlated features, and only 1 of the 2 was accepted. The least absolute shrinkage and selection operator (LASSO) method was then used to select the most robust predictive radiomics features with good reproducibility and association with treatment response in the training cohort.^21^ This method minimizes the sum of residues’ squares, with the sum of the absolute values of the selected features’ coefficients being less than a tuning parameter (λ). As λ becomes smaller, the coefficients of more features were shrunk to 0. To avoid overfitting, the number of selected features should be less than one-tenth of cases in the training set. Features with nonzero coefficients were then subsequently used to build the radiomics signature and were normalized using the *z* scores of the training and validation cohort (eMethods in the Supplement). Finally, a radiomics signature of each patient with GC was calculated with a linear combination of the final selection of features and multiplied by their normalized coefficients to obtain a radiomics signature.

### Performance and Clinical Usefulness of the Radiomics Signature

The performance, discrimination, calibration, and clinical usefulness of the radiomics signature are described in the eMethods in the Supplement. Briefly, the area under receiver operating characteristics curve (AUC) was used to assess the discriminative power of the radiomics signature and the Mann-Whitney *U* test to evaluate the correlation between the radiomics signature and treatment response status. Calibration curves were plotted to assess the calibration of the radiomics signature, accompanied with the Hosmer-Lemeshow test. To quantify the discrimination performance of the radiomics signature, Harrell’s C index was measured. Decision curve analysis (DCA) was performed to quantify the clinical utility of the radiomics signature from a simulated cohort data of the training and validation data set to assess the ability of the derived radiomics signature to differentiate potential respondents from nonrespondents. An ROC plot was used to illustrate net benefit from neoadjuvant therapy, defined as the proportion of true respondents minus the proportion of false respondents, displayed as functions of the risk threshold.

### Statistical Analysis

We used R version 3.3.1 (R Project for Statistical Computing) and SPSS statistical software version 20.0 (IBM Corp) for statistical analyses. A 2-sided *P* < .05 was considered statistically significant.

## Results

### Clinicopathological Characteristics of the Investigated Patients

The entire study cohort comprised of 323 patients (242 [74.1%] men; median [range] age, 58 [24-82] years) with locally advanced GC, with 250 patients (77.4%) in the training cohort and 73 (22.6%) in the validation cohort. Most patients were diagnosed with stage III locally advanced GC at the time of diagnosis (246 [76.2%]), with 13 (4.0%) with stage IIA; 29 (9.0%) with stage IIB; and 35 (10.8%) with stage IVA.

The mean number of neoadjuvant chemotherapy cycles was 3 (range, 2-4) cycles. The median time to surgery of the training and validation cohort was 3 (range, 2-4) weeks. There was no significant difference in the pretreatment clinical characteristics between the training and validation cohort, except for the percentage of patients with calcium levels in the reference range (210 [84.0%] vs 71 [97.3%]; *P* = .003), AST levels in the reference range (171 [68.4%] vs 62 [84.9%]; *P* = .045), activated partial thromboplastin time in the reference range (182 [72.8%] vs 64 [87.7%]); *P* = .009), and histological grade (eg, high or moderate grade: 48 [19.2%] vs 28 [38.4%]; *P* = .001) (eTable in the Supplement).

### Association of Patients’ Characteristics to Neoadjuvant Treatment

The number of respondents (TRG 1a-1b) and nonrespondents (TRG 2-3) in the training cohort was 122 (48.8%) and 128 (51.2%), respectively; in the training cohort, the number of respondents and nonrespondents was 40 (54.8%) and 33 (45.2%), respectively (Table 1). Our analyses showed that no patient characteristics were associated with neoadjuvant chemotherapy response, including commonly used clinical, serological, and radiographic variables, such as age, carcinoembryonic antigen level, CA199 level, CA724 level, tumor location, clinical tumor stage, clinical node stage, and clinical TNM stage in both the training and validation cohorts.

**Table 1.** Association of Patient Characteristics With Treatment Response in the Training and Validation Cohorts

Characteristic	Training cohort, No. (%)		*P* value	Validation cohort, No. (%)		*P* value
	Respondents	Nonrespondents		Respondents	Nonrespondents	
Sex						
Female	31 (20.0)	33 (27.3)	.95	8 (20.0)	9 (27.3)	.46
Male	91 (80.0)	95 (72.7)		32 (80.0)	24 (72.7)	
Age, median (range), y	57 (29-74)	58 (24-78)	.49	58 (30-82)	58 (28-71)	.10
Height, median (range), cm	167 (161-171)	165 (159-170)	.11	170 (162.25-172)	169 (162-173)	.97
Weight, median (range), kg	60 (54-69.75)	58.75 (52-65)	.11	63.5 (58-69.75)	61.5 (56-79.5)	.10
WBC						
Median (range), /μL	6.48 (5.52-7.51)	6.09 (5.14-8.19)	.61	5.74 (4.98-7.11)	5.45 (4.81-7.27)	.80
In reference range	112 (97.5)	112 (93.9)	.27	39 (97.5)	31 (93.9)	.87
Outside reference range	10 (2.5)	16 (6.1)		1 (2.5)	2 (6.1)	
Neutrophils						
Median (range), /μL	4.03 (3.25-4.81)	3.82 (2.85-5.06)	.50	3.41 (2.68-4.35)	3.31 (3.09-4.62)	.27
In reference range	112 (87.5)	109 (81.8)	.10	35 (87.5)	27 (81.8)	.73
Outside reference range	10 (12.5)	19 (18.2)		5 (12.5)	6 (18.2)	
Lymphocytes						
Median (range), /μL	1.71 (1.31-2.33)	1.72 (1.30-2.19)	.53	1.65 (1.38-2.15)	1.57 (1.21-1.8)	.08
In reference range	107 (82.5)	114 (84.8)	.74	33 (82.5)	28 (84.8)	.79
Outside reference range	15 (17.5)	14 (15.2)		7 (17.5)	5 (15.2)	
Monocytes						
Median (range), /μL	0.51 (0.32-0.61)	0.53 (0.44-0.58)	.78	0.36 (0.27-0.45)	0.35 (0.28-0.43)	.67
In reference range	100 (97.5)	104 (93.9)	.88	39 (97.5)	31 (93.9)	.87
Outside reference range	22 (2.5)	24 (6.1)		1 (2.5)	2 (6.1)	
RBC						
Median (range), ×10^6^/μL	4.42 (4.00-4.86)	4.35 (3.89-4.67)	.10	4.28 (3.95-4.64)	4.54 (3.86-4.79)	.24
In reference range	72 (42.5)	69 (60.6)	.42	17 (42.5)	20 (60.6)	.12
Outside reference range	50 (57.5)	59 (39.4)		23 (57.5)	13 (39.4)	
Hemoglobin						
Median (range), g/dL	126.50 (106.75-139.51)	127.55 (108.25-138.00)	.89	131.05 (99.25-144.00)	130.12 (106.04-145.50)	.74
In reference range	53 (52.5)	60 (51.5)	.59	21 (52.5)	17 (51.5)	.93
Outside reference range	69 (47.5)	68 (48.5)		19 (47.5)	16 (48.5)	
Platelets						
Median (range), ×10^3^/μL	262.50 (204.75-322.50)	268.54 (216.02-322.01)	.48	276.10 (236.25-332.08)	241.06 (217.50-314.01)	.14
In reference range	85 (67.5)	80 (69.7)	.23	27 (67.5)	23 (69.7)	d
Outside reference range	37 (32.5)	48 (30.3)		13 (32.5)	10 (30.3)	
Mean platelet volume						
Median (range), fL	9.49 (8.45-10.43)	9.61 (8.31-10.58)	.95	9.50 (8.63-10.78)	9.22 (8.80-10.25)	.81
In reference range	75 (65.0)	80 (66.7)	.87	26 (65.0)	22 (66.7)	.88
Outside reference range	47 (35.0)	48 (33.3)		14 (35.0)	11 (33.3)	
Calcium						
Median (range), mg/dL	2.24 (2.16-2.33)	2.27 (2.15-2.34)	.69	2.31 (2.21-2.36)	2.25 (2.19-2.34)	.14
In reference range	101 (97.5)	109 (97.0)	.61	39 (97.5)	32 (97.0)	>.99
Outside reference range	21 (2.5)	19 (3.0)		1 (2.5)	1 (3.0)	
Magnesium						
Median (range), mg/dL	0.91 (0.86-0.96)	0.91 (0.86-0.95)	.66	0.88 (0.81-0.93)	0.88 (0.79-0.93)	.76
In reference range	113 (87.5)	121 (81.8)	.54	35 (87.5)	27 (81.8)	.73
Outside reference range	9 (12.5)	7 (18.2)		5 (12.5)	6 (18.2)	
ALT						
Median (range), U/L	14.10 (10.55-20.35)	13.54 (10.05-19.21)	.31	14.12 (10.25-19.50)	15.02 (12.35-19.54)	.52
In reference range	99 (82.5)	104 (87.9)	.98	33 (82.5)	29 (87.9)	.76
Outside reference range	23 (17.5)	24 (12.1)		7 (17.5)	4 (12.1)	
AST						
Median (range), U/L	17.45 (14.74-21.59)	17.15 (14.15-20.53)	.45	19.51 (16.35-22.30)	21.01 (19.27-26.14)	.10
In reference range	83 (82.5)	88 (87.9)	.90	33 (82.5)	29 (87.9)	.76
Outside reference range	39 (17.5)	40 (12.1)		7 (17.5)	4 (12.1)	
Albumin						
Median (range), g/dL	40.25 (36.91-43.33)	39.91 (36.89-42.22)	.38	42.84 (38.52-44.07)	41.37 (38.20-43.01)	.25
In reference range	64 (72.5)	61 (60.6)	.45	29 (72.5)	20 (60.6)	.28
Outside reference range	58 (27.5)	67 (39.4)		11 (27.5)	13 (39.4)	
Globulin						
Median (range), g/dL	27.85 (25.07-30.63)	28.31 (25.06-30.68)	.80	23.85 (21.35-26.68)	24.15 (21.60-27.28)	.58
In reference range	116 (85.0)	119 (81.8)	.72	34 (85.0)	27 (81.8)	.72
Outside reference range	6 (15.0)	9 (18.2)		6 (15.0)	6 (18.2)	
BUN						
Median (range), mg/dL	5.19 (4.30-6.42)	5.07 (4.24-6.06)	.29	5.53 (4.83-6.77)	5.30 (4.29-6.29)	.27
In reference range	111 (95.0)	113 (90.9)	.48	38 (95.0)	30 (90.9)	.82
Outside reference range	11 (5.0)	15 (9.1)		2 (5.0)	3 (9.1)	
Creatinine						
Median (range), mg/dL	75.41 (64.12-87.43)	73.40 (65.05-83.08)	.45	62.11 (50.78-77.62)	54.02 (47.51-70.19)	.23
In reference range	93 (60.0)	99 (48.5)	.84	24 (60.0)	16 (48.5)	.33
Outside reference range	29 (40.0)	29 (51.5)		16 (40.0)	17 (51.5)	
PT, median (range)						
Median (range), s	11.40 (10.81-12.15)	11.24 (10.71-11.89)	.18	11.54 (10.93-11.90)	11.32 (10.91-11.85)	.54
In reference range	99 (87.5)	106 (93.9)	.73	35 (87.5)	31 (93.9)	.60
Outside reference range	23 (12.5)	22 (6.1)		5 (12.5)	2 (6.1)	
APTT						
Median (range), s	25.70 (23.31-29.73)	26.12 (24.03-28.97)	.75	27.35 (25.22-30.08)	26.20 (24.23-28.05)	.16
In reference range	92 (85.0)	90 (90.9)	.37	34 (85.0)	30 (90.9)	.68
Outside reference range	30 (15.0)	38 (9.1)		6 (15.0)	3 (9.1)	
Fasting blood glucose						
Median (range), mg/dL	3.48 (2.85-4.32)	3.42 (2.92-4.24)	.85	3.27 (2.74-4.06)	3.38 (2.74-4.11)	.85
In reference range	85 (75.0)	87 (72.7)	.77	30 (75.0)	24 (72.7)	.83
Outside reference range	37 (25.0)	41 (27.3)		10 (25.0)	9 (27.3)	
CEA						
Median (range), ng/mL	2.45 (1.48-6.41)	3.35 (1.66-7.51)	.22	2.06 (1.09-4.87)	2.34 (1.12-6.25)	.59
In reference range	86 (77.5)	81 (69.7)	.23	31 (77.5)	23 (69.7)	.45
Outside reference range	36 (22.5)	47 (30.3)		9 (22.5)	10 (30.3)	
CA199						
Median (range)	8.69 (3.28-20.78)	9.52 (6.94-24.29)	.78	8.69 (4.75-22.07)	7.12 (4.39-22.47)	.98
In reference range	101 (82.5)	86 (84.8)	.08	33 (82.5)	28 (84.8)	.79
Outside reference range	21 (17.5)	42 (15.2)		7 (17.5)	5 (15.2)	
CA724						
Median (range)	3.18 (1.28-10.24)	3.99 (2.39-12.82)	.74	3.02 (1.97-8.04)	5.24 (1.65-18.77)	.60
In reference range	77 (70.0)	78 (51.5)	.72	28 (70.0)	17 (51.5)	.11
Outside reference range	45 (30.0)	50 (48.5)		12 (30.0)	16 (48.5)	
cT stage^a^						
2	6 (<0.1)	5 (6.1)	.35	0	2 (6.1)	.46
3	56 (42.5)	73 (42.4)		17 (42.5)	14 (42.4)	
4a	46 (42.5)	40 (36.4)		17 (42.5)	12 (36.4)	
4b	14 (15.0)	10 (15.2)		6 (15.0)	5 (15.2)	
cN stage^a^						
0	15 (10.0)	7 (21.2)	.23	4 (10.0)	7 (21.2)	.28
1	22 (17.5)	29 (21.2)		7 (17.5)	7 (21.2)	
2	39 (45.0)	35 (21.2)		18 (45.0)	7 (21.2)	
3a	38 (15.0)	50 (21.2)		6 (15.0)	7 (21.2)	
3b	8 (12.5)	7 (15.2)		5 (12.5)	5 (15.2)	
cTNM stage^a^						
IIA	6 (0.0)	5 (6.1)	.07	0	2 (6.1)	.11
IIB	14 (7.5)	5 (21.2)		3 (7.5)	7 (21.2)	
III	88 (77.5)	108 (57.6)		31 (77.5)	19 (57.6)	
IVA	14 (15.0)	10 (15.2)		6 (15.0)	5 (15.2)	
Tumor location						
Upper third	48 (22.5)	57 (24.2)	.68	9 (22.5)	8 (24.2)	.55
Middle third	40 (62.5)	40 (51.5)		25 (62.5)	17 (51.5)	
Lower third	34 (15.0)	31 (24.2)		6 (15.0)	8 (24.2)	
Lauren type						
Intestinal	46 (42.5)	37 (33.3)	.23	17 (42.5)	11 (33.3)	.14
Diffuse	34 (35.0)	42 (24.2)		14 (35.0)	8 (24.2)	
Mixed	42 (22.5)	49 (42.4)		9 (22.5)	14 (42.4)	
WHO histological grade						
High/moderate	29 (40.0)	19 (36.4)	.25	16 (40.0)	12 (36.4)	.83
Low	51 (37.5)	63 (48.5)		15 (37.5)	16 (48.5)	
Signet ring/mucinous	42 (22.5)	46 (15.1)		9 (22.5)	5 (15.1)	
*ERBB2* status						
Positive	5 (65.0)	14 (69.7)	.08	3 (7.5)	2 (6.1)	.76
Negative	76 (7.5)	84 (6.1)		26 (65.0)	23 (69.7)	
NA	41 (27.5)	30 (24.2)		11 (27.5)	8 (24.2)	

Abbreviations: ALT, alanine aminotransferase; APTT, activated partial thromboplastin time; AST, aspartate aminotransferase; BUN, blood urea nitrogen; CEA, carcinoembryonic antigen; NA, not applicable; PT, prothrombin time; RBC, red blood cells; WBC, white blood cells; WHO, World Health Organization.

SI conversions: To convert albumin and globulin to grams per liter, multiply by 10; ALT and AST to microkatals per liter, multiply by 0.0167; BUN to millimoles per liter, multiply by 0.357; calcium to millimoles per liter, multiply by 0.25; CEA to micrograms per liter, multiply by 1.0; creatinine to micromoles per liter, multiply by 88.4; glucose to micromoles per liter, multiply by 0.0555; hemoglobin to grams per liter, multiply by 10.0; lymphocytes, monocytes, neutrophils, and WBC to ×10^9^ per liter, multiply by 0.001; magnesium to millimoles per liter, multiply by 0.4114; platelets to ×10^9^ per liter, multiply by 1.0; and RBC to ×10^12^ per liter, multiply by 1.0.

^a^ Classified using the clinical stage of the eighth edition of the American Joint Committee on Cancer TNM classification.

### Radiomics Features Extraction and Reproducibility

Radiomics techniques were then used to identify features that could differentiate between respondents and nonrespondents for neoadjuvant therapy. Initially, a total of 7477 two-dimensional radiomics features were extracted from the volume of interest of each patient. Among them, 2233 features were considered to have satisfactory intra-observer and interobserver reproducibility (ie, ICC >0.75). After omitting feature pairs with high correlations, 378 features from each patient were used for further selection.

### Radiomics Feature Selection and Radiomics Signature Building

**Figure 2.**
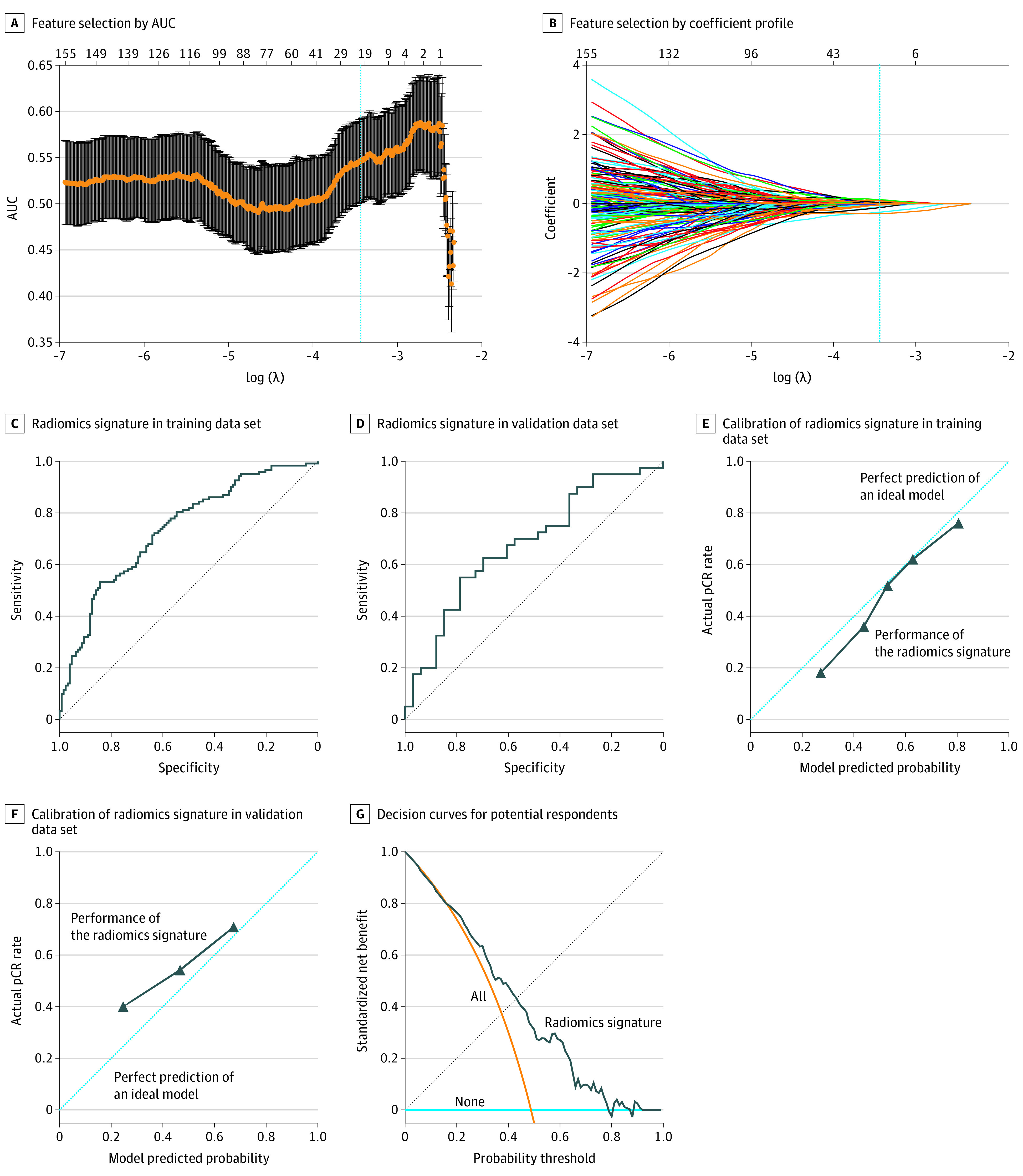
Radiomics Features Selection, Validation, and Clinical Utility of the Proposed Radiomics Signature A and B, Feature selection using the least absolute shrinkage and selection operator binary logistic regression model. This method minimizes the sum of residues’ squares, with the sum of the absolute values of the selected features coefficients being less than a tuning parameter (λ). A, The area under the receiver operating characteristic curve (AUC) was plotted vs log(λ). The vertical blue line represents the chosen parameter. B, Each colored line represents the coefficient of each feature. A vertical blue line was drawn at the λ selected, where 20 features had nonzero coefficients. C and D, Receiver operating characteristic curves of the radiomics signature in the training cohort (C) and validation cohort (D). E and F, Plots depicting the calibration of the radiomics signature in terms of agreement between predicted and observed respondents in the training cohort (E) and validation cohort (F). G, Decision curves for predicting potential respondents for neoadjuvant chemotherapy. The orange line represents the assumption that all patients will respond to neoadjuvant treatment, and the horizontal blue line represents the assumption that no patient will respond to treatment.

Using the LASSO logistic regression model in the training cohort (Figure 2A and B), 20 independent features (Table 2) from the 378 robust radiomics features were identified. They were used in the formula that appears in Figure 1 to build the radiomics signature.

**Table 2.** Characteristics of Each Feature Extracted

Radiomics feature	Mean (SD)	*P* value	AUC
GLCM_IMC2	0.788 (0.111)	.04	0.568 (0.496-0.639)
db1_3_GLDM_DNN	0.279 (0.075)	.06	0.563 (0.491-0.633)
db7_1_GLCM_sum_average	35.586 (7.437)	.002	0.605 (0.534-0.675)
db7_2_GLDM_LDLGLE	0.052 (0.032)	.05	0.569 (0.497-0.640)
db10_2_GLCM_correlation	−0.035 (0.073)	.27	0.536 (0.464-0.608)
db10_2_GLCM_max_p	0.060 (0.066)	.60	0.527 (0.455-0.599)
db10_4_GLSZM_LAHGLE	592.661 (720.345)	.18	0.540 (0.469-0.612)
sym8_3_GLDM_SDHGLE	96.343 (35.021)	.23	0.553 (0.482-0.625)
coif3_1_GLCM_clu_shade	−1121.563 (1396.325)	.07	0.561 (0.490-0.633)
coif5_1_GLSZM_LALGLE	3.475 (13.330)	.11	0.517 (0.445-0.589)
coif5_3_GLDM_LDE	5.621 (4.252)	.08	0.534 (0.463-0.606)
bior2.2_3_GLCM_correlation	0.011 (0.161)	.08	0.574 (0.503-0.645)
bior2.8_1_GLCM_inv_var	0.357 (0.077)	.07	0.564 (0.493-0.635)
rbio3.1_2_skewness	1.878 (2.486)	.07	0.555 (0.484-0.627)
rbio3.1_4_GLRLM_LRHGLE	389.906 (329.081)	.16	0.538 (0.467-0.610)
rbio3.7_1_skewness	−1.749 (1.554)	.007	0.607 (0.537-0.677)
rbio3.7_1_GLDM_SDHGLE	58.439 (25.697)	.04	0.569 (0.498-0.640)
rbio3.7_4_GLSZM_LAHGLE	2868.406 (9857.904)	.10	0.526 (0.454-0.598)
rbio3.7_4_GLDM_LDHGLE	787.964 (330.238)	.09	0.471 (0.399-0.544)
rbio5.5_2_GLCM_correlation	0.118 (0.129)	.17	0.546 (0.474-0.618)

Abbreviations: AUC, area under the curve; clu_shade, cluster shade; GLCM, gray level co-occurrence matrix; IMC, informational measure of correlation; GLDM, gray level dependence matrix; DNN, dependence nonuniformity normalized; GLRLM, gray level run length matrix; GLSZM, gray level size zone matrix; LAHGLE, large area high gray level emphasis; LALGLE, large area low gray level emphasis; LDE, large dependence emphasis; LDHGLE, large dependence high gray level emphasis; LDLGLE, large dependence low gray level emphasis; LRHGLE, long run high gray level emphasis; SDHGLE, small dependence high gray level emphasis.

### Response Prediction Performance of the Radiomics Signature

The AUCs of the radiomics signature for discriminating respondents from nonrespondents was 0.736 (95% CI, 0.675-0.798) in the training cohort (Figure 2C) and 0.679 (95% CI, 0.554-0.803) in the validation cohort (Figure 2D). Mann-Whitney *U* test showed good correlations between radiomics signature and treatment response in the training and validation cohort. To determine the agreement between the predicted and observed pathological response, the radiomics signature was calibrated using calibration plots to illustrate the goodness of fit (Figure 2E) in the training cohort and validated in the external validation cohort (Figure 2F). The Hosmer-Lemeshow test yielded a nonsignificant statistic, which suggested a small difference from perfect fit between the predicted and observed treatment response.

**Figure 3.**
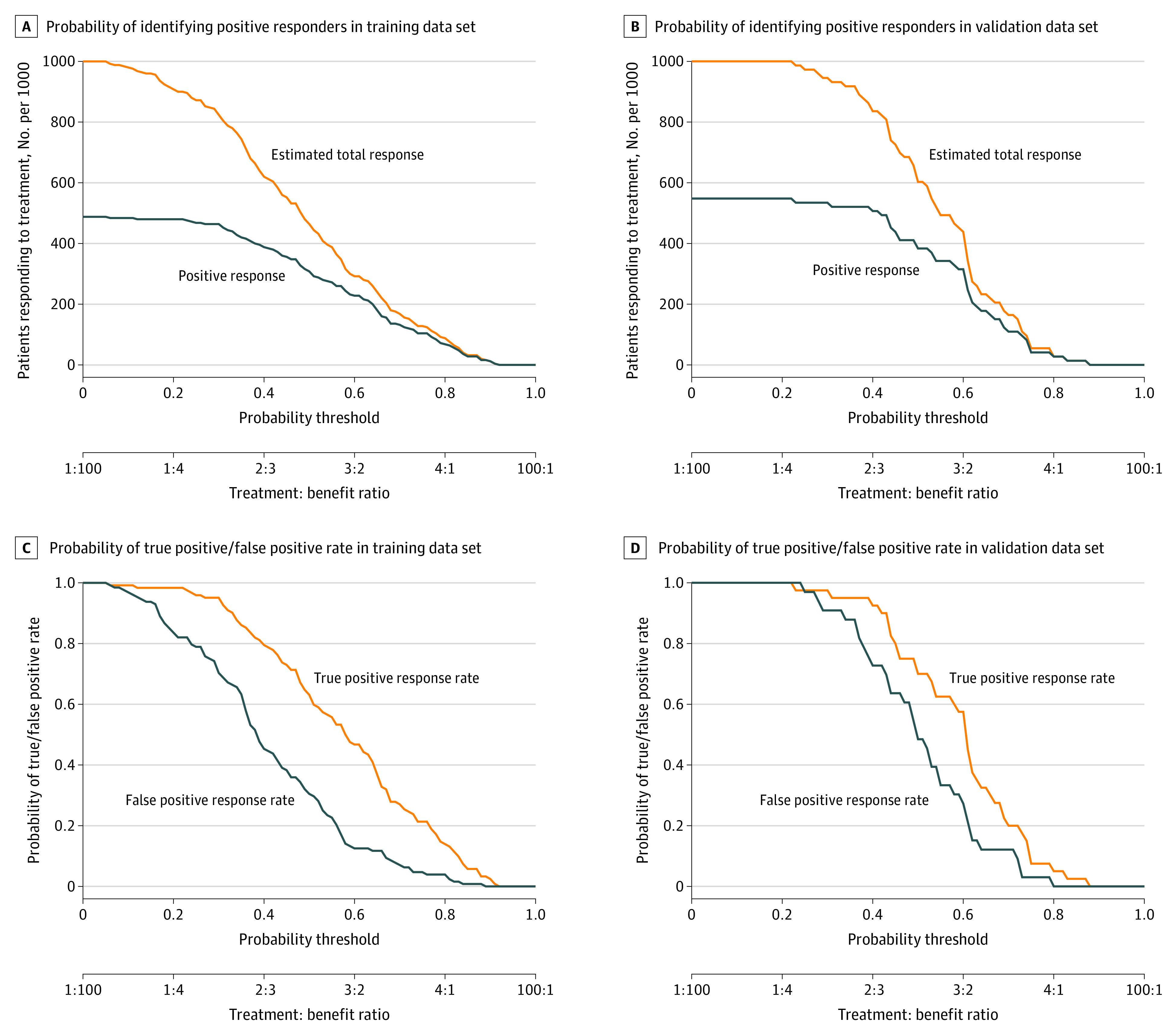
Predictive Assessment of Respondents From a Simulated Model Comprising 1000 Patients With Locally Advanced Gastric Cancer A and B, The closer the curves, the higher the probability that the radiomics signature would identify positive responders from a total estimated number of responders. The threshold value represents the value after which the rate of misdiagnosis would be lowest, thereby providing an optimal treatment to benefit ratio for the patient. The blue line represents the total number of patients who would be considered as having a true-positive response (tumor regression grade 1a-1b) for each threshold. C and D, Probability assessment using the radiomics signature to differentiate between true-positive (tumor regression grade 1a-1b) and false-positive (tumor regression grade 2-3) responders. The farther apart the curves are, the greater the probability that the model would differentiate between the 2 types of responders.

The DCA plot (Figure 2G) showed that the radiomics signature could predict patients’ response for probability thresholds ranging between 0.20 and 0.75. To further assess the clinical utility of the radiomics signature, a simulated model comprised of 1000 locally advanced GC cases of the training and validation cohorts was built to more accurately identify patients with potential positive response. Our findings showed a probability threshold of 0.6 in both cohorts (Figure 3A and B) was optimal to identify patients who would have positive response from neoadjuvant chemotherapy. Furthermore, the probability threshold of 0.5 to 0.6 in both the training and validation cohorts (Figure 3C and D) was optimal for differentiating true-positive responders from false-positive responders. Thus, using the threshold value of the radiomics signature on the DCA and ROC plots, a reliable individualized prediction of the response of a patient with locally advanced GC patient to neoadjuvant chemotherapy could be obtained.

## Discussion

In this large multicentered study comprised of locally advanced GC patients, no clinicopathological characteristics were significantly associated with treatment response. Radiomics was able to bridge radiology and histopathology by using postoperative pathological findings as a reference standard to identify potential responders for neoadjuvant chemotherapy. The extracted 20 features were used to generate a radiomics signature that was then used to identify an individualized threshold value from DCA and ROC plots, based on which potential respondents to neoadjuvant therapy could be identified.

Previous studies have shown that clinicopathological characteristics (such as age, signet-ring cell type histology, and microsatellite status) were associated with neoadjuvant response.^22,23,24,25^ However, the characteristics identified were not consistent across all reports. Also, they were single-center studies with small sample sizes, and their findings were not externally validated. We hypothesize the lack of association between clinicopathological characteristics and treatment response in our present study could be because of intratumoral heterogeneity, such as underlying mutation characteristics or cell cycle regulating pathways, which could be more strongly associated with treatment response than pretreatment clinicopathological characteristics given that neoadjuvant therapy, being a systemic treatment, can reshape tumor-immune signaling and the tumor microenvironment.^26,27,28^ Thus, intratumoral heterogeneity could be reflected, to a certain extent, by the unique textural and spatial gray level patterns of the extracted radiomics features in the pretreatment in vivo CT images. However, these theoretical assumptions are yet to be fully elucidated via radiogenomics and multi-omics studies.

The data used for this study were derived from 3 institutions, and heterogeneity in clinical and pathological data or CT imaging collection and interpretation at the different institutions may have existed. To overcome this, 3 experienced radiologists (with 8, 10, and 13 years of experience) performed intra-observer and interobserver reproducibility reassessment, for which only features with good reproducibility (ie, ICC >0.75) were selected to build the signature. Furthermore, using the radiomics score combined with the threshold on the DCA (Figure 2G) and ROC plots (Figure 3), the net benefit for suggesting neoadjuvant chemotherapy to a patient (ie, the proportion of true positive minus the proportion of false positive weighted by the relative consequence of false-positive and false-negative results as compared with a treat-all or treat-none) could be estimated.

To our knowledge, this is the first study to develop and externally validate a radiomics signature for patients with locally advanced GC. It could be used to offer evidence-based guidance, rather than solely relying on clinical judgment, for multidisciplinary consultation between radiologists and oncologists to predict pretreatment response and offer justifiable individualized therapeutic options to these patients. Also, for those patients predicted as having a high probability of stable or progressive disease (TRG 2 or 3), timely alternative curative-intent treatment approaches could be offered to improve their treatment and survival outcomes. Thus, the strength of this study is using existing routine diagnostic CT imaging and a validated method, without additional cost to the patient, to predict their treatment response at diagnosis.

### Limitations

This study has limitations. First, the retrospective nature of the analyses bears incoherent biases that could have affected the study findings, but we believe that these were partially solved via intra-observer and interobserver reproducibility assessments and DCA. Second, CT images were retrieved from different hospitals that could have different acquisition protocols that could have, to a certain extent, affected the radiomics analyses. Despite selecting patients who were similar in the general process of CT examination to reduce such a limitation, different protocols are implemented in the clinical practice at different institutions, and this could in turn potentially improve the reproducibility of the multi-institutional study findings. Third, despite this being a multicenter validated study with a large cohort of patients with locally advanced GC and considering the heterogeneous nature of GC among different populations and ethnicities, additional validation in larger prospective cohorts of patients is still warranted to further confirm the clinical significance of the proposed radiomics signature.

## Conclusions

Using a large cohort of patients with locally advanced GC who received neoadjuvant treatment, we proposed and externally validated a radiomics signature to differentiate between potential respondents and nonrespondents to neoadjuvant chemotherapy. This signature could be used as a clinical treatment decision aid for oncologists to predict neoadjuvant chemotherapy response, thereby allowing timely planning of effective personalized treatments to potential nonrespondents. We expect the findings of this study to be used as a baseline for larger cohorts or prospective studies to improve individualized predictions of which patients with GC will respond to neoadjuvant treatment.

## Notes

Supplement. **eMethods. **Supplementary Methods
eReferences.
**eTable.** Baseline Comparison of the Clinicopathological Characteristics Between the Training and Validation Cohorts

## References

[1] Ferlay J, Soerjomataram I, Dikshit R, . Cancer incidence and mortality worldwide: sources, methods and major patterns in GLOBOCAN 2012. Int J Cancer. 2015;136(5):E359-E386. doi:10.1002/ijc.29210 25220842

[2] Wang FH, Shen L, Li J, . The Chinese Society of Clinical Oncology (CSCO): clinical guidelines for the diagnosis and treatment of gastric cancer. Cancer Commun (Lond). 2019;39(1):10. doi:10.1186/s40880-019-0349-9 30885279 PMC6423835

[3] Paoletti X, Oba K, Burzykowski T, ; GASTRIC (Global Advanced/Adjuvant Stomach Tumor Research International Collaboration) Group. Benefit of adjuvant chemotherapy for resectable gastric cancer: a meta-analysis. JAMA. 2010;303(17):1729-1737. doi:10.1001/jama.2010.534 20442389

[4] Ajani JA, Winter K, Okawara GS, . Phase II trial of preoperative chemoradiation in patients with localized gastric adenocarcinoma (RTOG 9904): quality of combined modality therapy and pathologic response. J Clin Oncol. 2006;24(24):3953-3958. doi:10.1200/JCO.2006.06.4840 16921048

[5] Cunningham D, Allum WH, Stenning SP, ; MAGIC Trial Participants. Perioperative chemotherapy versus surgery alone for resectable gastroesophageal cancer. N Engl J Med. 2006;355(1):11-20. doi:10.1056/NEJMoa055531 16822992

[6] Walsh TN, Noonan N, Hollywood D, Kelly A, Keeling N, Hennessy TP. A comparison of multimodal therapy and surgery for esophageal adenocarcinoma. N Engl J Med. 1996;335(7):462-467. doi:10.1056/NEJM199608153350702 8672151

[7] Zhang ZX, Gu XZ, Yin WB, Huang GJ, Zhang DW, Zhang RG. Randomized clinical trial on the combination of preoperative irradiation and surgery in the treatment of adenocarcinoma of gastric cardia (AGC)—report on 370 patients. Int J Radiat Oncol Biol Phys. 1998;42(5):929-934. doi:10.1016/S0360-3016(98)00280-6 9869212

[8] Herrmann K, Ott K, Buck AK, . Imaging gastric cancer with PET and the radiotracers 18F-FLT and 18F-FDG: a comparative analysis. J Nucl Med. 2007;48(12):1945-1950. doi:10.2967/jnumed.107.044867 18006614

[9] Ott K, Herrmann K, Krause BJ, Lordick F. The value of PET imaging in patients with localized gastroesophageal cancer. Gastrointest Cancer Res. 2008;2(6):287-294. 19259277 PMC2632563

[10] Napieralski R, Ott K, Kremer M, . Combined GADD45A and thymidine phosphorylase expression levels predict response and survival of neoadjuvant-treated gastric cancer patients. Clin Cancer Res. 2005;11(8):3025-3031. doi:10.1158/1078-0432.CCR-04-1605 15837757

[11] Jia Y, Ye L, Ji K, . Death-associated protein-3, DAP-3, correlates with preoperative chemotherapy effectiveness and prognosis of gastric cancer patients following perioperative chemotherapy and radical gastrectomy. Br J Cancer. 2014;110(2):421-429. doi:10.1038/bjc.2013.712 24300973 PMC3899757

[12] Peng H, Dong D, Fang MJ, . Prognostic value of deep learning PET/CT-based radiomics: potential role for future individual induction chemotherapy in advanced nasopharyngeal carcinoma. Clin Cancer Res. 2019;25(14):4271-4279. doi:10.1158/1078-0432.CCR-18-3065 30975664

[13] Liu A, Wang Z, Yang Y, . Preoperative diagnosis of malignant pulmonary nodules in lung cancer screening with a radiomics nomogram. Cancer Commun (Lond). 2020;40(1):16-24. doi:10.1002/cac2.12002 32125097 PMC7163925

[14] Antunovic L, De Sanctis R, Cozzi L, . PET/CT radiomics in breast cancer: promising tool for prediction of pathological response to neoadjuvant chemotherapy. Eur J Nucl Med Mol Imaging. 2019;46(7):1468-1477. doi:10.1007/s00259-019-04313-8 30915523

[15] Zheng J, Kong J, Wu S, . Development of a noninvasive tool to preoperatively evaluate the muscular invasiveness of bladder cancer using a radiomics approach. Cancer. 2019;125(24):4388-4398. doi:10.1002/cncr.32490 31469418

[16] Huang YQ, Liang CH, He L, . Development and validation of a radiomics nomogram for preoperative prediction of lymph node metastasis in colorectal cancer. J Clin Oncol. 2016;34(18):2157-2164. doi:10.1200/JCO.2015.65.9128 27138577

[17] Liu Z, Zhang XY, Shi YJ, . Radiomics analysis for evaluation of pathological complete response to neoadjuvant chemoradiotherapy in locally advanced rectal cancer. Clin Cancer Res. 2017;23(23):7253-7262. doi:10.1158/1078-0432.CCR-17-1038 28939744

[18] Amin MB, Edge S, Greene F, , eds. AJCC Cancer Staging Manual. 8th ed. Springer; 2017.

[19] World Medical Association. World Medical Association Declaration of Helsinki: ethical principles for medical research involving human subjects. JAMA. 2013;310(20):2191-2194. doi:10.1001/jama.2013.281053 24141714

[20] Becker K, Langer R, Reim D, . Significance of histopathological tumor regression after neoadjuvant chemotherapy in gastric adenocarcinomas: a summary of 480 cases. Ann Surg. 2011;253(5):934-939. doi:10.1097/SLA.0b013e318216f449 21490451

[21] Peduzzi P, Concato J, Kemper E, Holford TR, Feinstein AR. A simulation study of the number of events per variable in logistic regression analysis. J Clin Epidemiol. 1996;49(12):1373-1379. doi:10.1016/S0895-4356(96)00236-3 8970487

[22] Jiang L, Ma Z, Ye X, Kang W, Yu J. Clinicopathological factors affecting the effect of neoadjuvant chemotherapy in patients with gastric cancer. World J Surg Oncol. 2021;19(1):44. doi:10.1186/s12957-021-02157-x 33563277 PMC7874458

[23] Rajabnejad A, Vaida F, Valasek M, . Predictors and significance of histologic response to neoadjuvant therapy for gastric cancer. J Surg Oncol. 2021;123(8):1716-1723. doi:10.1002/jso.26458 33735448

[24] Ikoma N, Estrella JS, Blum Murphy M, . Tumor regression grade in gastric cancer after preoperative therapy. J Gastrointest Surg. 2021;25(6):1380-1387. doi:10.1007/s11605-020-04688-2 32542556 PMC11957322

[25] Cai Z, Rui W, Li S, . Microsatellite status affects tumor response and survival in patients undergoing neoadjuvant chemotherapy for clinical stage III gastric cancer. Front Oncol. 2020;10:614785. doi:10.3389/fonc.2020.614785 33384963 PMC7770160

[26] Li Z, Gao X, Peng X, . Multi-omics characterization of molecular features of gastric cancer correlated with response to neoadjuvant chemotherapy. Sci Adv. 2020;6(9):eaay4211. doi:10.1126/sciadv.aay4211 32133402 PMC7043923

[27] Xu HY, Xu WL, Wang LQ, Chen MB, Shen HL. Relationship between p53 status and response to chemotherapy in patients with gastric cancer: a meta-analysis. PLoS One. 2014;9(4):e95371. doi:10.1371/journal.pone.0095371 24740294 PMC3989310

[28] Yeh YS, Chen YT, Tsai HL, . Predictive value of ERCC1, ERCC2, and XRCC expression for patients with locally advanced or metastatic gastric cancer treated with neoadjuvant mFOLFOX-4 chemotherapy. Pathol Oncol Res. 2020;26(2):1105-1116. doi:10.1007/s12253-019-00666-5 31077069

