# Supplementary material for: Development and Validation of a Computed Tomography–Based Radiomics Signature to Predict Response to Neoadjuvant Chemotherapy for Locally Advanced Gastric Cancer
Source: JAMA Netw Open. 2021 Aug 19;4(8):e2121143. doi: 10.1001/jamanetworkopen.2021.21143 (PMC8377567; doi:10.1001/jamanetworkopen.2021.21143)
Supplement: Supplement. — eMethods. Supplementary Methods eReferences. eTable. Baseline Comparison of the Clinicopathological Characteristics Between the Training and Validation Cohorts [file jamanetwopen-e2121143-s001.pdf]

Supplemental Online Content

Wang W, Peng Y, Feng X, et al. Development and validation of a computed tomography–based radiomics signature to predict response to neoadjuvant chemotherapy for locally advanced gastric cancer. *JAMA Netw Open.* 2021;4(8):e2121143. doi:10.1001/jamanetworkopen.2021.21143

eMethods. Supplementary Methods

eReferences.

eTable. Baseline Comparison of the Clinicopathological Characteristics Between the Training and Validation Cohorts

This supplemental material has been provided by the authors to give readers additional information about their work.

© 2021 Wang W et al. *JAMA Network Open.*

eMethods. Supplementary Methods

1. Acquisition of computed tomography (CT) images

All patients underwent abdominal and pelvic contrast-enhanced CT examination within 1 week before neoadjuvant therapy at their respective hospitals. CT images were obtained from an 8-slice (GE Lightspeed Ultra 8, GE Healthcare, Hino, Japan) or a 64-slice multi-detector row CT (LightSpeed VCT, GE Medical system, Milwaukee, Wis). The parameters of the images were as follows: 120 kV; 130 mAs; rotation time of 0.4-/0.6s; detector collimation of 8×0.625 mm or 64×0.625 mm; field of view of 350 mm × 350 mm; matrix, 512 × 512. For dynamic contrast-enhanced CT imaging, about 90-100 mL of iodinated contrast material (Ultravist 370, Bayer Schering Pharma, Berlin, Germany) was injected into the patients' antecubital vein at a rate of 3.0 to 3.5 mL/s with a pump injector (Ulrich CT Plus 150, Ulrich Medical, Ulm, Germany). The arterial phase images were obtained after a 20s delay following the intravenous injection of the contrast material and portal venous phase images were obtained after 45s of delay. Both arterial and portal venous phase CT images were reconstructed with a section thickness of 5 mm. The CT images were retrieved using the picture archiving and communication system (PACS; Carestream, Canada) at each institution.

2. CT-images feature extraction

Tumor segmentations were performed using portal venous phase CT images as portal venous phase is better distinguishing features between the tumor mass and adjacent normal gastric tissue.

© 2021 Wang W et al. *JAMA Network Open.*

Region of interest (ROI) was manually delineated along the outline of the entire visible tumor, excluding the gastric lumen, using the ITK-SNAP software (http://www.itksnap.org/). Intraluminal air, necrotic area, vessel shadow, pericolic fat were also meticulously excluded from the contours.

After integrating the ROI of the volume of interest (VOI), the largest manually delineated ROI slice was chosen from each VOI. Before feature extraction, the ROI was resampled using a pixel size of 1 mm × 1 mm. Hounsfield units (HU) in all the images were resampled into 100 bins with HU from –200 to 600 HU, with a fixed bin size of 8 HU. Then, a 2-dimensional radiomics feature extraction was performed on the retrieved CT images using an in-house software with algorithms implemented in Matlab 2016a (Mathworks, Natick, USA). The CT images of gastric cancer (GC) lesions were resampled with a pixel size of 1mm×1mm using linear interpolation, then, separately normalized with min-max normalization to convert the pixels from -200HU to 600 HU into a range of [1, 100] of integral intensities.

3. Feature generation:

A series of first-order statistics, shape features and texture features were generated from the image with and without being filtered.

3.1 First-order statistics

First-order statistics describe the distribution of voxel intensities within the CT image.

3.2.Shape-based features

These features are descriptors of the two-dimensional size and shape of the tumor region.

3.3.Texture features

Textural features describe patterns or the spatial distribution of pixel intensities. They were calculated from the following matrix:

(1) Gray-Level Co-Occurrence Matrix (GLCM)

(2) Gray-Level Run-Length matrix

(3) Gray Level Size Zone Matrix (GLSZM)

(4) Gray Level Dependence Matrix (GLDM)

(5) Neighbouring Gray Tone Difference Matrix (NGTDM)

When calculating texture features, the ROI was resampled using a bin number of 25. The formula of features was in https://pyradiomics.readthedocs.io/en/latest/ or 'Image biomarker standardization initiative - feature definitions'[1].

3.4. Filter features

These features are first-order statistics and texture features extracted from filtered images. The image was filtered in the following ways, respectively:

(1) Image filtration:

© 2021 Wang W et al. *JAMA Network Open.*

A Laplacian of Gaussian spatial band-pass filter (∇2G) was used to derive image features at different spatial scales by turning the filter parameter between 1.0 and 2.5 (1.0, 1.5, 2.0, 2.5).

The Laplacian of Gaussian filter (∇2G) distribution is given by

$$\nabla^2 G(x,y) = \frac{-1}{\pi\sigma^4}\left(1 - \frac{x^2+y^2}{2\sigma^2}\right)e^{-\frac{x^2+y^2}{2\sigma^2}}$$

x and *y* denote the spatial coordinates of the pixel, and σ is the value of the filter parameter.

From the image filtrated, the first order statistic and texture features were extracted. A prefix ‘L_G’ and the number representing parameters were added to the features’ names.

(2) Wavelet features:

These features were extracted from an image after decomposing by different wavelets. Different functions (high-pass or low-pass, represented by H or L) on a different scale (*X*, *Y*) were represented by a number from 1 to 4 (LL, LH, HL, HH).

Wavelet decomposition should follow the formula:

$$X'(\mathrm{i},\mathrm{j}) = \sum_{p}^{N_x}\sum_{q}^{N_y} H_x(p)H_y(q)X(i+p,j+q)$$

The following wavelets were used: db1, db4, db7, db10, sym7, sym8, coif1, coif3, coif5, bior2.2, bior2.8, bior3.1, bior3.7, bior4.4, bior5.5, bior6.8, rbio2.2, rbio2.8, rbio3.1, rbio3.7, rbio4.4, rbio5.5, rbio6.8. From the image reconstructed, the first order statistic and texture features were extracted. A prefix including wavelet name and the number representing function was added to the features’ names.

© 2021 Wang W et al. *JAMA Network Open.*

4. Feature normalization

All selected features by the least absolute shrinkage and selection operator (LASSO) method were normalized with the z-scores of the training and validation cohort using the mean and standard deviation derived from the features of the training cohort The formula used is as follows:

$$X' = \frac{X - \bar{X}}{SD}$$

where X represent the value of each selected feature in a patient, while X' represent the corresponding normalized value. $\bar{X}$ represent the mean of the values of the feature and *SD* is the standard deviation in the training cohort. The mean and standard deviation of each feature are shown in **Table 2**.

5. Performance of radiomics signature

In the training cohort, the area under the receiver operating characteristics (ROC) curve (AUC) was used to assess the discriminative power of the radiomics signature. The Mann-Whitney U test was also applied to evaluate the correlation between the radiomics signature and treatment response status in both the training and validation cohorts.

The C-index was calculated as a measure of the goodness of fit for the radiomics signature, equaling to the ROC curve. Calibration was used to investigate the consistency between observed outcomes and predicted values which examine how well the percentage of observed outcome matches the percentage of predicted outcome over deciles of predicted risk. This is preferably

© 2021 Wang W et al. *JAMA Network Open.*

reported graphically with predicted outcome probabilities (on the x-axis) plotted against observed outcome frequencies (on the y-axis). The calibration curve was drawn by plotting ^P on the x-axis and $P_C = [1 + exp - (\gamma_0 + \gamma_1 L)]^{-1}$ on the y-axis, where P_C is the actual probability, L = logit (^P), ^P is the predicted probability, γ_0 is the corrected intercept, and γ_1 is the slope estimates.

6. Clinical usefulness

The decision curve analysis (DCA), proposed by Vickers et al.[2], as a validated method to quantify the clinical utility of a predictive model, was used for estimating the clinical usefulness of the DCA. The net benefit was calculated by subtracting the proportion of all patients who are false positive from the proportion who are truly positive, weighting by the relative harm of forgoing treatment compared with the negative consequences of unnecessary treatment. A clinical impact plot was drawn to estimate the number of cases that would be asserted as positive response for each threshold[3]. Further, a simulated cohort using data from the training and validation cohort was built to assess the ability of the derived radiomics score to identify respondents from non-respondents. The ROC plot was used to show the constituents of standardized net benefit, namely the true- and false-positive response rates to neoadjuvant chemotherapy, where the true- and false-positive fractions are displayed as functions of the risk threshold.

© 2021 Wang W et al. *JAMA Network Open.*

eReferences.

1. Zwanenburg A, Vallieres M, Abdalah MA, et al. The Image Biomarker Standardization Initiative: Standardized Quantitative Radiomics for High-Throughput Image-based Phenotyping. *Radiology.* May 2020;295(2):328-338.
2. Vickers AJ, Elkin EB. Decision curve analysis: a novel method for evaluating prediction models. *Medical decision making : an international journal of the Society for Medical Decision Making.* Nov-Dec 2006;26(6):565-574.
3. Kerr KF, Brown MD, Zhu K, Janes H. Assessing the Clinical Impact of Risk Prediction Models With Decision Curves: Guidance for Correct Interpretation and Appropriate Use. *J Clin Oncol.* Jul 20 2016;34(21):2534-2540.

© 2021 Wang W et al. *JAMA Network Open.*

eTable. Baseline Comparison of the Clinicopathological Characteristics Between the Training and Validation Cohorts

Characteristics	Training cohort (*n* = 250)		Validation cohort (*n* = 73)		*P*
Sex					0.69
Female	64	25.6%	17	23.3%	
Male	186	74.4%	56	76.7%	
Age (years) median (range)	58 (24-78)		58 (28-82)		0.13
Height (cm) Median (range)	166 (159-171)		169 (162-173)		0.23
Weight (kg) Median (range)	59 (52-69.8)		62 (56-79.5)		0.21
WBC					0.10
Normal	224	89.6%	70	95.9%	
Abnormal	26	10.4%	3	4.1%	
Neutrophils					0.43
Normal	221	88.4%	62	84.9%	
Abnormal	29	11.6%	11	15.1%	
Lymphocytes					0.28
Normal	221	88.4%	61	83.6%	
Abnormal	29	11.6%	12	16.4%	
Monocytes					0.05
Normal	204	81.6%	70	95.9%	
Abnormal	46	18.4%	3	4.1%	
RBC					0.39
Normal	141	56.4%	37	50.7%	
Abnormal	109	43.6%	36	49.3%	
HGB					0.30
Normal	113	45.2%	38	52.1%	
Abnormal	137	54.8%	35	47.9%	
PLT					0.69
Normal	165	66.0%	50	68.5%	
Abnormal	85	34.0%	23	31.5%	
MPV					0.56
Normal	155	62.0%	48	65.8%	
Abnormal	95	38.0%	25	34.2%	

© 2021 Wang W et al. *JAMA Network Open.*

Ca					0.003
Normal	210	84.0%	71	97.3%	
Abnormal	40	16.0%	2	2.7%	
Mg					0.06
Normal	234	93.6%	62	84.9%	
Abnormal	16	6.4%	11	15.1%	
ALT					0.47
Normal	203	81.2%	62	84.9%	
Abnormal	47	18.8%	11	15.1%	
AST					0.045
Normal	171	68.4%	62	84.9%	
Abnormal	79	31.6%	11	15.1%	
ALB					0.06
Normal	125	50.0%	49	67.1%	
Abnormal	125	50.0%	24	32.9%	
GLOB					0.05
Normal	235	94.0%	61	83.6%	
Abnormal	15	6.0%	12	16.4%	
BUN					0.37
Normal	224	89.6%	68	93.2%	
Abnormal	26	10.4%	5	6.8%	
CRE					0.18
Normal	192	76.8%	40	54.8%	
Abnormal	58	23.2%	33	45.2%	
PT					0.09
Normal	205	82.0%	66	90.4%	
Abnormal	45	18.0%	7	9.6%	
APTT					0.009
Normal	182	72.8%	64	87.7%	
Abnormal	68	27.2%	9	12.3%	
Fbg					0.40
Normal	172	68.8%	54	74.0%	
Abnormal	78	31.2%	19	26.0%	
CEA					0.25
Normal	167	66.8%	54	74.0%	
Abnormal	83	33.2%	19	26.0%	
CA199					0.12
Normal	187	74.8%	61	83.6%	
Abnormal	63	25.2%	12	16.4%	
CA724					0.96

© 2021 Wang W et al. *JAMA Network Open.*

Normal	155	62.0%	45	61.6%	
Abnormal	95	38.0%	28	38.4%	
cT stage[a]					0.07
2	11	4.4%	2	2.7%	
3	129	51.6%	31	42.5%	
4a	86	34.4%	29	39.7%	
4b	24	9.6%	11	15.1%	
cN stage[a]					0.32
0	22	8.8%	11	15.1%	
1	51	20.4%	14	19.2%	
2	74	29.6%	25	34.2%	
3a	88	35.2%	13	17.8%	
3b	15	6.0%	10	13.7%	
cTNM stage[a]					0.84
IIA	11	4.4%	2	2.7%	
IIB	19	7.6%	10	13.7%	
III	196	78.4%	50	68.5%	
IVA	24	9.6%	11	15.1%	
Tumor location					0.30
Upper third	105	42.0%	17	23.3%	
Middle third	80	32.0%	42	57.5%	
Lower third	65	26.0%	14	19.2%	
Lauren type					0.37
Intestinal	83	33.2%	28	38.4%	
Diffuse	76	30.4%	22	30.1%	
Mixed	91	36.4%	23	31.5%	
WHO histological grade					0.001
High/moderate	48	19.2%	28	38.4%	
Low	114	45.6%	31	42.5%	
Signet ring/Mucinous	88	35.2%	14	19.1%	
HER-2 status					0.69
Positive	19	7.6%	5	6.8%	
Negative	160	64.0%	49	67.2%	
NA	71	28.4%	19	26.0%	

[a]Classified using the 8th edition of the American Joint Committee on Cancer (AJCC) TNM classification;

Abbreviations: WBC, white blood cells; RBC, red blood cells; HGB, hemoglobin; PLT, platelets; MPV, mean platelet volume; Ca, calcium; Mg, magnesium; ALT, alanine aminotransferase; ALB, albumin;

© 2021 Wang W et al. *JAMA Network Open.*

GLOB, globulin; BUN, blood urea nitrogen; CRE, creatinine; PT, prothrombin time; APTT, activated partial thromboplastin time; Fbg, fasting blood glucose; CEA, carcinoembryonic antigen; T, tumor depth; N, node; M, metastasis; NA, not available;

© 2021 Wang W et al. *JAMA Network Open.*
